# Comparison of Myocardial Viability by Cardiac Magnetic Resonance Imaging and Echocardiography in Patients With Myocardial Infarction: An Observational Cross-Sectional Study

**DOI:** 10.7759/cureus.99290

**Published:** 2025-12-15

**Authors:** Ajay Bhatta, Arun Maskey, Nirmal P Neupane, Anupam Bista, Sudip Lamsal, Astha Wagle, Susmita Gyawali, Nirmal Ghimire

**Affiliations:** 1 Department of Medicine, Geta Hospital, Kailali, NPL; 2 Department of Cardiology, Shahid Gangalal National Heart Centre, Kathmandu, NPL; 3 Department of Radiodiagnosis, Shahid Gangalal National Heart Centre, Kathmandu, NPL; 4 Department of Medicine, Bharatpur Hospital, Chitwan, NPL; 5 Department of Cardiology, National Academy of Medical Sciences, Kathmandu, NPL; 6 Department of Pathology, KIST Medical College and Teaching Hospital, Kathmandu, NPL; 7 Central Department of Public Health, Institute of Medicine - Tribhuvan University, Kathmandu, NPL; 8 Department of Internal Medicine, Nepal Police Hospital, Kathmandu, NPL

**Keywords:** coronary artery disease, echocardiography, late gadolinium enhancement, mri, viability

## Abstract

Background and aim

Coronary artery disease (CAD) is one of the major causes of morbidity and mortality worldwide. Following myocardial infarction (MI), patients may develop significant ventricular dysfunction, and many others may harbor a burden of residual coronary disease, for which viability assessment of the suspected myocardial territory may be needed. Thus, this study aimed to assess the viability of myocardium by cardiac magnetic resonance imaging (MRI) in patients with MI and compare it with wall motion abnormality in echocardiography.

Methods

An observational cross-sectional study was conducted at Shahid Gangalal National Heart Centre and Bir Hospital, Kathmandu, Nepal. A total of 90 patients with a history of MI who underwent cardiac MRI were enrolled from May 12, 2023, to May 11, 2024. Echocardiography was analyzed for wall motion abnormalities and left ventricular ejection fraction (LVEF), while cardiac MRI was analyzed for wall motion abnormalities, LVEF, and late gadolinium enhancement (LGE). Myocardial segments were divided into those with LGE ≤50% (viable) and those with LGE >50% (non-viable). Wall motion abnormality by echocardiography and LGE by cardiac MRI were assessed for each segment and compared.

Results

Out of a total of 1,530 myocardial segments in 90 patients, 431 segments showed LGE on cardiac MRI, out of which 38 segments had LGE ≤50%, and 393 segments had LGE >50%. Most of the myocardial segments with LGE on cardiac MRI were in the left anterior descending (LAD) artery territory. All 38 segments that showed LGE ≤50% showed hypokinesia on echocardiogram. None of these segments showed akinesia or dyskinesia on echocardiogram. The majority of the segments with LGE >50% showed akinesia on echocardiogram, followed by hypokinesia and dyskinesia. None of the segments with LGE >50% had normal wall motion.

Conclusion

Cardiac MRI is an accurate and reliable tool to assess cardiac volumes, function, and LGE, thus determining the extent and severity of infarcted myocardium. Wall motion abnormalities by echocardiography, based on their severity, can also help to predict which involved myocardium may be viable and thus benefit in resource-limited settings.

## Introduction

Coronary artery disease (CAD) is one of the major causes of morbidity and mortality worldwide, among which myocardial infarction (MI) is the most severe presentation [1]. The invention of reperfusion strategies, including primary percutaneous coronary intervention (PCI) for the management of acute ST-elevation MI (STEMI), has significantly improved mortality, but the morbidity associated with post-MI heart failure (HF) is still significant [2,3]. Following MI, patients may develop significant ventricular dysfunction and residual CAD, for which viability evaluation of the suspected myocardial territory is required [4]. The involved myocardial tissue has shown improved function and contractility following revascularization [5]. The modalities used for assessing myocardial viability include echocardiography, single-photon emission computed tomography (SPECT), positron emission tomography (PET), and cardiac magnetic resonance imaging (MRI) [6]. Echocardiography establishes hypokinetic, akinetic, or dyskinetic myocardium [7]. Cardiac MRI offers a unique advantage for assessment of myocardial viability due to its ability to visualize scar and its extent with the help of late gadolinium enhancement (LGE) [8].

Since the availability of cardiac MRI, which is a gold standard, is limited, most of the time echocardiography remains the only imaging modality to evaluate regional wall motion abnormalities (RWMAs). Thus, this study aims to compare myocardial viability by cardiac MRI and echocardiography, thereby highlighting the importance of echocardiography in assessing myocardial viability in a resource-limited setting.

## Materials and methods

Study design, setting, and participants

This was an observational cross-sectional study. The study was conducted in the Department of Cardiology at Shahid Gangalal National Heart Centre (SGNHC) and Bir Hospital, Kathmandu, Nepal. Patients with MI who met the inclusion criteria were included in the study from May 12, 2023, to May 11, 2024.

Sample size

The minimum required sample size was calculated using the formula: sample size $n = \frac{Z^2 \cdot p \cdot q}{d^2}$, where p is the percentage of viable myocardial segments (i.e., LGE <50%) detected by cardiac MRI and is equal to 36.2%, q = 1 - p, i.e., 63.8%, d is the precision and is equal to 0.05, and Z is the confidence interval and is equal to 1.96. There are no data assessing myocardial viability by cardiac MRI from Nepal. A study by Shah et al., carried out in 50 Indian patients, was published in the Cureus journal in 2022 [9]. This study showed that LGE was seen in 378 out of 850 (44.5%) myocardial segments. Out of 378 segments detected on LGE, 137 (36.2%) segments showed <50% LGE. Our aim was to assess viable myocardium (LGE <50%). Therefore, n = 677 myocardial segments were required to estimate LGE <50%. As LGE was observed in 44.5% of total myocardial segments, we assumed that 1,522 segments needed to be observed. As 17 segments of myocardium were studied per patient, it was estimated that 1,522/17 ≈ 90 patients needed to be enrolled.

Sampling method

Non-probability consecutive sampling method was used to include the patients in the study. All patients diagnosed with MI who meet the predefined inclusion and exclusion criteria during the study period will be consecutively enrolled until the required sample size is achieved. Each eligible patient will undergo both cardiac MRI and echocardiography for the assessment of myocardial viability.

Inclusion and exclusion criteria

The inclusion criteria for the study were as follows: a history of MI, a left ventricular ejection fraction (LVEF) of less than 50%, age greater than 18 years, and willingness to provide informed consent.

The exclusion criteria for the study were patients with a history of PCI or coronary artery bypass grafting, those with cardiac implantable electronic devices, patients with an estimated glomerular filtration rate (eGFR) <30 mL/min/1.73 m², patients with arrhythmias including atrial fibrillation or atrial flutter, and hemodynamically unstable patients.

Operational definitions

*Wall Motion Abnormalities* ​​​​​

Normal wall motion: systolic thickening of >30%; hypokinesia: systolic thickening of <30%; akinesia: systolic thickening of <10%; dyskinesia: no appreciable systolic thickening, with systolic movement away from the center of the left ventricle (LV) [10].

*Myocardial Viability* ​​​​​

Viable myocardium: LGE <50% on cardiac MRI and non-viable myocardium: LGE >50% on cardiac MRI [8].

Procedural details

Patients admitted to the medical ward or those from the Outpatient Department were enrolled in the study after obtaining informed written consent. Medical records, which included age, gender, electrocardiography (ECG), and transthoracic echocardiography, were evaluated. Patients with MI were taken for the study based on their clinical history, cardiac enzymes, ECG, or echocardiography. Transthoracic echocardiography was performed with a commercially available imaging system, the Philips Affiniti 50 Echocardiography machine (Philips Healthcare, Amsterdam, Netherlands), using a 2.5 MHz phased-array transducer. Cardiac chamber size, LVEF, and RWMA were assessed. Cardiac MRI was performed in eligible patients with a history of MI and reduced LVEF where immediate revascularization was not mandated (e.g., acute STEMI). Cardiac MRI was done on a 3-Tesla platform (Philips Ingenia 3T MRI machine; Philips Healthcare), and images were obtained both without and with contrast. LGE sequences were performed 10 minutes post-contrast after injection of 0.1 mmol/kg of gadolinium contrast. The following sequences were obtained: survey, transverse black blood image of the chest, vertical long axis (VLA), four-chamber (4C), and short-axis (SA) cine sequences; left ventricular outflow tract (LVOT) and three-chamber cine sequences; VLA, 4C, and SA STIR (short tau inversion recovery) images; delayed gadolinium enhancement PSIR (phase-sensitive inversion recovery) sequences in SA, 4C, and VLA planes. LV volumes and LVEF, along with RWMA, were assessed by cardiac MRI using a 17-segment model. RWMA were classified as normal, hypokinetic, akinetic, or dyskinetic. Transmurality of LGE was classified as 0%, 1-25%, 26-50%, 51-75%, and 76-100% in all segments. Segments with LGE ≤50% were considered viable. Wall motion abnormalities assessed by echocardiography were compared with LGE by cardiac MRI.

Ethical consideration

The Institutional Review Board of the National Academy of Medical Sciences, Bir Hospital, granted ethical approval (approval number: 894/2079/80). All study participants gave their informed consent to allow the use of anonymous personal and clinical data in research, which was used to ensure complete confidentiality.

Statistical analysis

Data were compiled, edited, and checked daily to maintain consistency. The data were collected in Microsoft Excel (Microsoft® Corp., Redmond, WA, USA). For statistical analysis, IBM SPSS Statistics for Windows, Version 27.0 (Released 2019; IBM Corp., Armonk, NY, USA) was used. Quantitative variables are expressed as mean ± SD, and categorical variables as frequency and percentage. Comparison of wall motion abnormality and LGE by cardiac MRI was performed, and the p-value was calculated. Furthermore, wall motion abnormality by echocardiography was compared with LGE by cardiac MRI, and the p-value was assessed. Fisher's exact test was employed to assess the statistical significance of the association. A p-value of <0.001 was considered statistically significant.

## Results

A total of 90 patients with MI were included in the study. Seventy-four (82.2%) patients were males, and 16 (17.8%) patients were females. The age of the patients ranged from 29 to 85 years, with a mean age of 60.2 ± 11.5 years. Among the 90 patients, 63 had a history of anterior wall MI (AWMI), 25 had inferior wall MI (IWMI), and two had non-ST elevation MI (NSTEMI). The mean LVEF of the patients was 34.49 ± 9%.

Of the 431 segments showing LGE, 343, 53, and 35 segments were in the left anterior descending (LAD), right coronary artery (RCA), and left circumflex (LCx) territories, respectively. Out of 38 segments that showed LGE ≤50%, 30 segments were in the LAD territory, and four segments were in the RCA and LCx territories each. Similarly, among 393 segments that showed LGE >50%, 313 segments were in the LAD territory, 49 segments in the RCA territory, and 31 segments in the LCx territory. Wall motion abnormality by cardiac MRI of the involved territory showed akinesia in 67 patients and hypokinesia in 23 patients. Similarly, wall motion abnormality by echocardiography showed akinesia in 57 patients, hypokinesia in 32 patients, and dyskinesia in one patient.

Among a total of 1,530 myocardial segments in 90 patients, 431 segments showed LGE on cardiac MRI. Out of the 431 segments that showed LGE, 38 segments showed LGE ≤50%, and 393 segments showed LGE >50%. None of the segments with LGE ≤50% showed akinesia on cardiac MRI. All akinetic segments showed LGE >50% on cardiac MRI. Among all hypokinetic segments on cardiac MRI, 38 segments had LGE ≤50%, and 52 segments had LGE >50%. All 38 segments that showed LGE ≤50% showed hypokinesia on echocardiography. None of the segments with LGE ≤50% showed akinesia or dyskinesia on echocardiography (Table 1).

**Table 1:** Comparison of wall motion abnormality by MRI with LGE on cardiac MRI LGE: late gadolinium enhancement; MRI: magnetic resonance imaging

Wall Motion Abnormality (MRI)	Number of Segments (n)		p-value
	LGE ≤ 50% (MRI)	LGE > 50% (MRI)	
Akinesia	0	341	<0.001
Hypokinesia	38	52	
Total	38	393	

Out of 393 segments with LGE >50%, the majority of the segments (299 segments, 76.1%) showed akinesia on echocardiography. Similarly, 87 segments (22.13%) showed hypokinesia, and the remaining seven segments (1.78%) showed dyskinesia (Table 2). This was statistically significant (p-value <0.001).

**Table 2:** Comparison of wall motion abnormality by echocardiogram with LGE on cardiac MRI LGE: late gadolinium enhancement; MRI: magnetic resonance imaging

Wall Motion Abnormality (Echocardiogram)	Number of Segments (n)		p-value
	LGE ≤ 50% (MRI)	LGE > 50% (MRI)	<0.001
Akinesia	0	299	
Dyskinesia	0	7	
Hypokinesia	38	87	
Total	38	393	

## Discussion

MI is one of the life-threatening coronary artery-related diseases [11]. LV dysfunction and resultant HF after an MI are ever-increasing [12]. As a consequence, these patients are at risk for decreased functional capacity and quality of life, hospitalization, and death [13]. Revascularization therapy, whenever appropriate, is the definite treatment post-MI [14]. Moreover, LV dysfunction is not always due to an irreversible scar, but can result from impairment in the function of viable but dysfunctional myocardium [15]. Prompt identification of this viable myocardium with the help of imaging modalities decreases the chances of morbidity and mortality later in life [16]. For the evaluation of viability, cardiac MRI is the gold standard, and, using a paramagnetic contrast agent (gadolinium), scar can be identified using LGE [17].

More than two-thirds of patients in our study were males, which was similar to the study done by Nowosielski et al., which included 52 patients, of whom 84.6% were males [18]. Another study by Gerber et al., which assessed LGE by cardiac MRI in 144 patients, had 90.28% (n = 130) males [19]. It has been observed that men are generally more predisposed to MI compared to women due to a combination of biological, lifestyle, and behavioral factors, which was also observed in our study. The mean age of the patients in our study was 60.2 ± 11.5 years. This finding was similar to the study by Gerber et al. and another study by Gardner et al., where the mean ages were 65 ± 11 years and 66 ± 11 years, respectively [19,20], which points to the fact that, with rising age, people are significantly more predisposed to MI due to age-related physiological changes and a higher prevalence of risk factors and comorbidities. The mean LVEF in our patient population was 34.49 ± 9%, which was similar to that reported in the study by Shah et al. [9].

**Figure 1:**
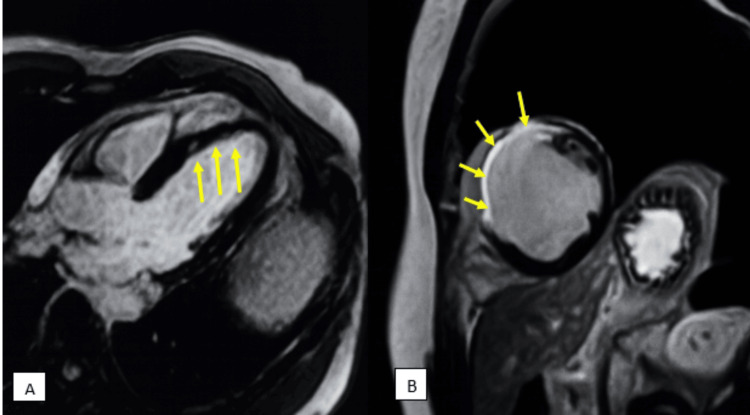
A) Late gadolinium enhancement showing sub-endocardial infarction, with <25% wall thickness enhancement in the septum in the left anterior descending territory (thus viable); B) Late gadolinium enhancement (short-axis view) showing >75% wall thickness enhancement, suggestive of transmural infarction (thus non-viable), in the left anterior descending territory

Out of a total of 1,530 myocardial segments examined among 90 patients, 431 segments (28.17%) showed LGE on cardiac MRI in our study. Among them, 38 (8.8%) segments showed LGE ≤50%, and the remaining 393 (91.2%) segments showed LGE >50%, as shown in Figure 1.

Comparing these findings to those in the study by Shah et al. showed that a higher proportion of segments showed LGE in their study (44.5%) [9]. Their study also had a higher proportion of myocardial segments with LGE >50%, similar to ours. In contrast, the study by Aggarwal et al. showed a higher percentage (80%) of myocardial segments with LGE <50%, and only 20% of the segments showed LGE >50% [21]. None of the segments with LGE >50% showed normal wall motion, which was statistically significant (p < 0.001). The majority of myocardial segments showing LGE on cardiac MRI were in the LAD territory in our study, which is also comparable to the study done by Shah et al. [9]. The most common area for an MI is the LAD artery, which supplies blood to the LV and is the most frequent site of blockage, as also seen in our study. All 38 myocardial segments with LGE ≤50% showed hypokinesia on echocardiography, and none of the segments showed akinesia or dyskinesia in our study. Similarly, among 393 segments with LGE >50%, most of the segments showed akinesia (299 segments, 76.1%), followed by hypokinesia (87 segments, 22.13%) and dyskinesia (7 segments, 1.78%), which was statistically significant (p < 0.001), as shown in Videos 1-3.

**Video 1:** Parasternal short-axis view in echocardiography showing hypokinetic inferior walls

**Video 2:** Parasternal short-axis view in echocardiography showing dyskinetic inferior walls

**Video 3:** Parasternal long-axis view in echocardiography showing akinetic inferolateral walls

Thus, our study highlights the use of echocardiography to predict viability, even in centres where cardiac MRI is not available. Not all akinetic walls are non-viable, and vice versa. Therefore, we compared wall motion abnormality by echocardiography with the gold standard test for viability, only to assess how well the echocardiogram performs compared with cardiac MRI. Viability testing is not always performed in existing standard practice, as it is time-consuming, bears additional cost, and is not available everywhere. Thus, our study highlights that simple echocardiography can also predict viability in patients who have suffered MI, before performing revascularization treatment.

There are some limitations of the study. Many guidelines highlight on assessment of myocardial viability in order to select appropriate candidates who would benefit from revascularization. Also, a consensus on multimodality imaging signifies that cardiac MRI and PET should be preferred when available. As PET is not yet available for viability testing in our country, we did cardiac MRI and compared it with echocardiography. In the study, most of the myocardial segments had LGE >50% probably because patients with severe wall motion abnormalities were preferentially subjected to viability testing by cardiac MRI. Further studies with a higher sample size and follow-up studies involving multiple centers are needed to confirm the findings.

## Conclusions

Cardiac MRI with LGE is one of the most reliable and accurate modalities for assessing cardiac volumes, mass, stroke volume, LVEF, wall motion abnormalities, and the extent, severity, and viability of the infarcted myocardium, which is of key importance for revascularization therapy. However, using the most commonly and easily available echocardiography to assess ventricular function and wall motion abnormalities can also guide us regarding which of the involved segments are likely viable prior to revascularization. This can be very useful in settings where cardiac MRI is not available.
